# Respiratory failure in cancer patients with influenza A (H1N1) is associated with poor prognosis

**DOI:** 10.1186/cc9642

**Published:** 2011-03-11

**Authors:** E Snyder, M Cardenas-Turanzas, C Perego, R Erfe, RC Chemaly, KP Price, JL Nates

**Affiliations:** 1 The University of Texas MD Anderson Cancer Center, Houston, TX, USA

## Introduction

During the spring of 2009, the influenza A (H1N1) virus emerged, resulting in an estimated 12,000 deaths in the United States. We aimed to describe the critically ill patients with cancer who developed 2009 H1N1 in a comprehensive cancer center.

## Methods

We conducted an observational study of patients >17 years of age with confirmed infection from 1 June 2009 to 30 April 2010. Data collected included demographics, clinical characteristics and outcomes.

## Results

A total of 9/2,629 adult patients (0.3%) admitted to the ICU were diagnosed with 2009 H1N1 influenza. Six patients were female, patient age ranged from 43 to 77 and all had hematological cancers. The ICU mortality rates were 16% for all-cause admissions and 78% for 2009 H1N1cases. The most frequent co-morbidities were obesity and hypertension. Eight patients were diagnosed with bilateral pneumonia. The median hospital length of stay (LOS) was 28 days (range 9 to 45) and ICU LOS was 8 days (range 2 to 31). The ventilation course of the nonsurvivors was characterized by progressive hypoxemia. At admission, 67% of patients had a PaO_2_/FiO_2 _less than 200; at day 7, 71% of patients, and at day 14, 100% of patients. The nonsurvivors (seven patients) received respiratory care by a range of ventilation mechanisms: patients received non-invasive mechanical ventilation, were intubated, and then utilized one or a combination of bilevel, pressure control and pressure support ventilation. One patient used high-frequency ventilation. Invasive ventilation lasted a median of 7 days (range 4 to 23). The survivors (two patients) received only supplemental oxygen. All patients were treated with antiviral medications and antibiotics. Four patients died from cardiac arrest and three patients died following life support therapy withdrawal. All nonsurvivors had DNR orders in place at death.

## Conclusions

At our center, the ICU mortality due to the 2009 H1N1 influenza was remarkably higher than that observed in patients with cancer without this infection. However, the number of patients developing the infection and requiring critical care was smaller than expected if considering we care for a population of patients with a high prevalence of immune suppression.

